# Carnosic Acid Mitigates Depression-Like Behavior in Ovariectomized Mice via Activation of Nrf2/HO-1 Pathway

**DOI:** 10.1007/s12035-022-03093-x

**Published:** 2022-11-04

**Authors:** Doaa M. Samy, Dalia Kamal Mostafa, Samar R. Saleh, Passainte S. Hassaan, Teshreen M. Zeitoun, Gamal A. G. Ammar, Nahed H. Elsokkary

**Affiliations:** 1 Department of Medical Physiology, Faculty of Medicine, University of Alexandria, Al-Mouassat Medical Campus, El Hadara, Alexandria, Egypt; 2 Department of Clinical Pharmacology, Faculty of Medicine, University of Alexandria, Alexandria, Egypt; 3 Department of Biochemistry, Faculty of Science, University of Alexandria, Alexandria, Egypt; 4 Bioscreening and Preclinical Trial Lab, Faculty of Science, University of Alexandria, Alexandria, Egypt; 5 Department of Histology and Cell Biology, Faculty of Medicine, University of Alexandria, Alexandria, Egypt; 6 Biotechnology Unit, Plant Production Department (PPD), Arid Lands Cultivation Research Institute (ALCRI), City of Scientific Research and Technological Applications (SRTA-City), New Borg El-Arab City, Alexandria, Egypt

**Keywords:** BDNF, Menopause, Oxidative stress, Serotonin, Thioredoxin-1, Tin protoporphyrin IX

## Abstract

The peri- and post-menopausal periods have been described as the “window of vulnerability” for the development of depressive symptoms that impair women activities and quality of life. The etiopathogenesis of these symptoms is multifactorial and may confer resistance to traditional antidepressants. Attention is now directed toward phytochemicals for their pleiotropic functions and safer profiles. This study investigated the possible perturbation of the nuclear factor erythroid 2–related factor 2 (Nrf2) signaling pathways as an underlying mechanism of post-ovariectomy depression and highlighted the potential benefits of carnosic acid (CA) on the associated behavioral, biochemical, and histopathological alterations. Female Balb/c mice were randomly assigned to be sham-operated or ovariectomized (OVX). After 3 weeks, OVX mice received either a vehicle, CA (20 mg/kg/day), or tin protoporphyrin IX (SnPP-IX; a heme oxygenase-1 (HO-1) inhibitor; 50 μmol/kg/day) for 3 weeks. Our findings revealed that OVX mice had depressive but not anxiety-like behavior. Suppressed Nrf2 and its downstream signaling, and augmented proinflammatory markers were observed in both the hippocampus and prefrontal cortex. CA treatment alleviated depressive behavior, induced the expression of Nrf2, HO-1, thioredoxin-1, and brain-derived neurotrophic factor, and enhanced serotonin levels. CA also suppressed oxidative stress, reduced TNF-α, IL-1β, and iNOS mRNA expression, and ameliorated OVX-induced histopathological changes. SnPP-IX aggravated post-OVX behavioral, neurobiochemical, and histological deteriorations, and reduced CA-protective effects. In conclusion, Nrf2/HO-1 signaling suppression and the associated proinflammatory state are key mechanisms in post-OVX depression. CA exerts multifaceted neuroprotection in OVX mice and represents a promising candidate for clinical evaluation as an antidepressant.

## Introduction

The prevalence of depression in women is noticeably increased during the transition to and after menopause. Studies revealed that women who enter perimenopause are twice as likely to have clinically significant depressive manifestations as those who have not yet made the menopausal transition [1, 2]. In fact, many cross-sectional and longitudinal studies have documented an increased risk of mood disorders in the perimenopausal period [3]. Furthermore, the inadequate responses to antidepressant drugs during menopause were linked to low estrogen level which was counteracted by estrogen therapy. Moreover, the traditional bilateral ovariectomy animal model exhibits a post-menopausal depressive-like state and is used successfully, not only as a proof of concept, but also to test the benefits of different treatment modalities [4]. Although a change in the serotonergic transmission secondary to estrogen deficiency has been implicated as an underlying factor, the precise mechanisms remain unknown [5–7].

It was previously shown that defective pathways of nuclear factor erythroid 2–related factor (Nrf2), the master redox-sensitive transcription factor involved in cellular defense against oxidative stress, could increase the susceptibility to anxiety and/or depressive-like behavior [8–10]. This was based on the evidence linking inflammation, oxidative stress, and depression, where an increase in proinflammatory cytokines and a reduction in antioxidant defenses were encountered [11, 12]. Moreover, many studies focused on uncovering the antioxidant and anti-inflammatory activities of some currently used antidepressant drugs. This may, in turn, raise interest in targeting Nrf2 as a possible approach to offer a better or complementary treatment to depressed patients. However, the molecular signaling cascade needs, first, to be more elucidated as Nrf2 can directly regulate the transcription of many downstream genes controlling the glutathione and thioredoxin antioxidant systems. Nrf2 also induces enzymes involved in the detoxification of exogenous and endogenous products, such as heme oxygenase (HO-1) and many others [13]. Intriguingly, Nrf2 has also been observed to affect brain-derived neurotrophic factor (BDNF) expression [14]. Given the essential roles of neurotrophins in neuronal plasticity and mood stability [15], pharmacological modulation of Nrf2 could have a promising pleiotropic benefit in depressive disorders.

In this context, carnosic acid (CA) which is a phenolic diterpene obtained from *Rosmarinus officinalis L*. (commonly known as “rosemary”) may be of particular interest. It is a well-known Nrf2 inducer exhibiting antioxidant and anti-inflammatory properties through the Nrf2/HO-1 axis [16]. The favorable role of HO-1 in neurodegenerative disorders, such as Alzheimer’s disease [17], encouraged us to investigate its link to depression behavior, especially that the neuroprotective effect of the antidepressant desipramine was related to increased HO-1 expression [18]. Furthermore, the benefits of some herbal medications on hippocampal cells have been linked to increased HO-1 activity [19]. In light of the previously reported antidepressant effect of either rosemary extract in depression and anxiety [20, 21], or CA itself in post-stroke or stress-induced depression [22, 23], the potential benefits of CA in peri/post-menopausal mood alteration merit to be investigated. This is fostered by the notion that CA can cross the blood–brain barrier (BBB) in a sufficient concentration to exert many cytoprotective effects [22].

Therefore, this study aimed to investigate the impact of the induction of menopause-like changes, by bilateral ovariectomy, on the central Nrf2 expression and the consequent perturbation of the oxidant and inflammatory milieu in cortical and hippocampal tissues in mice. In addition, the effects of CA supplementation on ovariectomy-induced anxiety and/or depressive-like behavior and the associated central biochemical and microstructural alterations were explored. Finally, we intended to study the impact of inhibiting HO-1 activity by tin protoporphyrin IX (SnPP-IX) and to analyze the specific role of the HO-1 pathway in the CA-proposed benefits, least present.

## Materials and Methods

### Experimental Animals

The study was conducted on 10-week-old female Balb/c mice with an average body weight of 25 g. Animals were purchased from and raised in the Animal House of the Physiology Department, Faculty of Medicine, Alexandria University. Animals were maintained under standard laboratory conditions and allowed free access to regular rodent chow and water. Mice were housed in groups of 8 in simple rectangular cages provided with sawdust as bedding material. Each cage was supplied with two bottles of water. Experimental procedures were carried out according to the NIH Guide for Care and Use of Laboratory Animals and complied with the ARRIVE guidelines. The study protocol was approved by the Research Ethics Committee, Faculty of Medicine, Alexandria University (IRB No: 00012098; FWA No: 00018699; approval No: 0305099).

### Chemicals and Reagents

Carnosic acid and tin protoporphyrin IX dichloride were purchased from AG Scientific, San Diego, and Frontier Scientific, Logan, UT, respectively. Reduced nicotinamide adenine dinucleotide phosphate (NADPH), glucose-6-phosphate, glucose-6-phosphate dehydrogenase, hemin, 5,5′-dithio-bis-2-nitrobenzoic acid, thiobarbituric acid (TBA), and pyrogallol have been obtained from Sigma-Aldrich (St. Louis, MO, USA). GENEzol™ Reagent was purchased from Geneaid, Taiwan, while the Viva cDNA synthesis kit and Taq qPCR Green Master Mix were purchased from Vivantis, Malaysia. Primers have been supplied by Invitrogen, Thermo Fisher Scientific, USA, while primary and secondary antibodies were obtained from Cell Signaling Technology, USA, and Biospes, China. Other chemicals were commercially available high-grade products.

### Surgical Procedures

Bilateral ovariectomy was done to all experimental animals except the sham-operated ones under intraperitoneal ketamine (100 mg/kg) and xylazine (10 mg/kg) anesthesia [24] to eliminate endogenous ovarian steroid production. The skin of the lower abdomen was shaved, local antiseptic was applied, and then a small transverse incision was made in the middle part of the abdomen. The ovaries were exteriorized with the associated fat pad and fallopian tubes. Hemostatic clamps were applied around the blood supply of the ovaries, and suture knots were made below it. The ovaries were then cut away and discarded. The muscle and skin layers were sutured and the wound was topically treated with betadine and then finally sprayed with an antibiotic. Sham-operated animals were exposed to the same surgical procedure except that the ovaries were exposed but not excised. After surgery, animals were kept in a warm place until full recovery. All animals were given 0.5 ml saline *i.p.* for rehydration and meloxicam 1 mg/kg *s.c.* for analgesia [25].

### Experimental Groups and Tissue Sampling

**Fig. 1:**
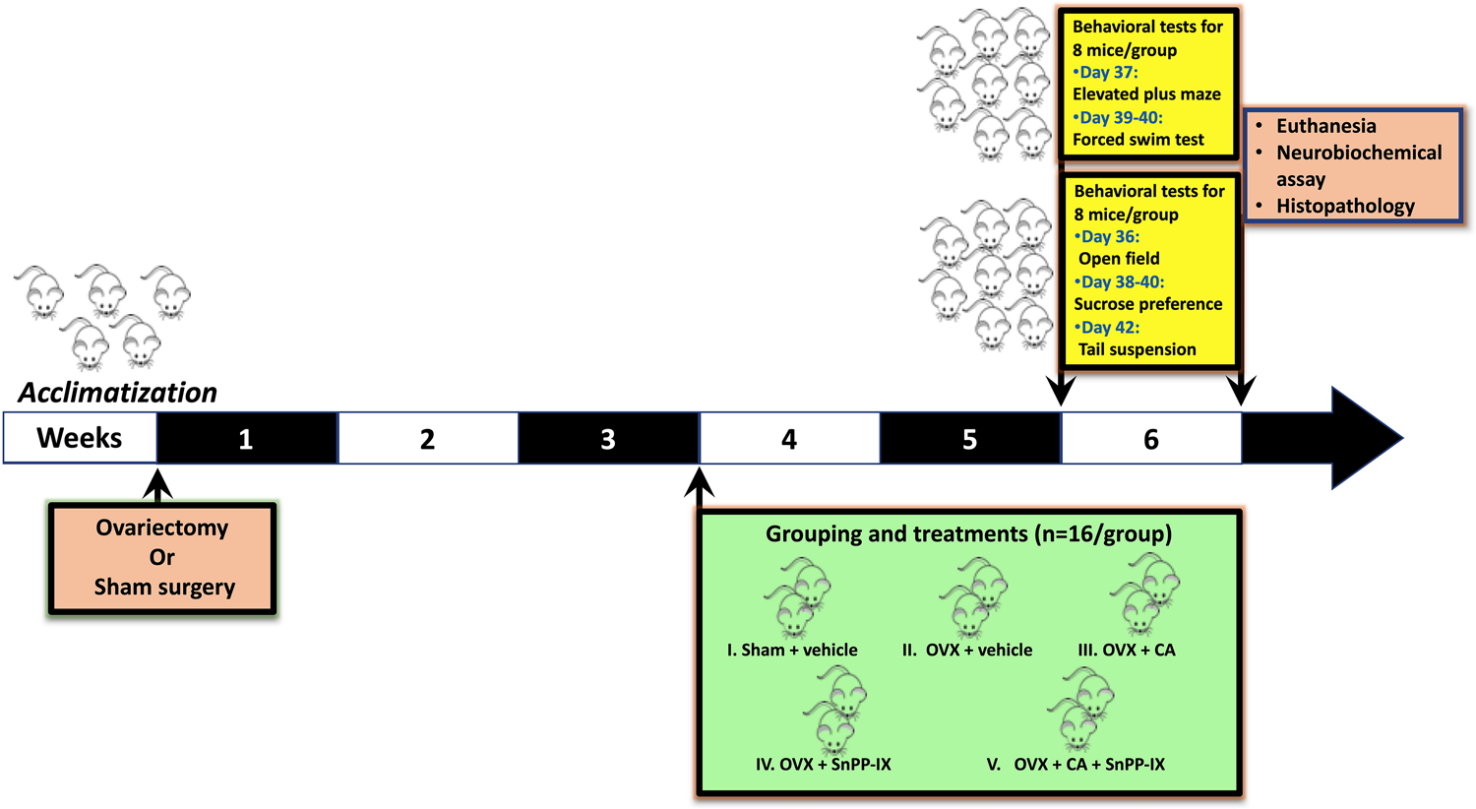
Schematic representation of the experimental design. OVX, ovariectomized; CA, carnosic acid; SnPP-IX, tin protoporphyrin IX

Three weeks following surgery, a total of 80 mice were randomly assigned into 5 experimental groups (*n* = 16 mice each) that received treatments or vehicles for a further 3 weeks as follows: control (sham-operated) mice were given both carboxy methyl cellulose (CMC) and phosphate-buffered saline (PBS) by oral gavage and *i.p.*, respectively, as vehicles (1 ml/100 g *b.w.*); ovariectomized (OVX) mice were given both vehicles as the sham-operated group; CA-treated OVX mice (OVX + CA) received 20 mg/kg/day of CA by oral gavage; tin protoporphyrin IX–treated OVX mice (OVX + SnPP-IX) received *i.p.* injection of 50 μmol/kg/day of SnPP-IX [26], and the combined-treated OVX mice (OVX + CA + SnPP-IX) received CA (20 mg/kg/day; *p.o.*) 15 min. following SnPP-IX injection (50 μmol/kg/day; *i.p.*). A schematic presentation of the study design is shown in Fig. 1.

The selected dose for CA was optimized to the average doses used in previous in vivo studies that reported cytoprotective effects via boosting Nrf2 activation without any toxicity manifestations [27–29]. CA was dissolved in 0.5% CMC and freshly prepared on the day of administration, while SnPP-IX was dissolved in 0.1 M NaOH, diluted in PBS, and titrated with 0.1 M HCl until the pH reached 7.4. Stock solution was weekly prepared in the dark, and aliquots were stored at − 80 °C and thawed immediately prior to *i.p.* injection.

Behavioral testing was conducted over the sixth-week post-surgery. At the end of the experiment, all animals were euthanized by decapitation under anesthesia using thiopental Na 50 mg/kg *i.p.* [24], and brain tissues were excised. The right hemispheres of a random set of 8 animals/group were processed for histopathological examination, while the hippocampi and the prefrontal cortices of the left hemispheres of this set, together with the two hemispheres of the rest of the animals, were dissected out and stored at − 80 °C for further biochemical analysis. The harvested tissues were divided into 4 subsets; the first one was used for western blot analysis, while the second one was used to assess HO-1 activity and oxidative stress markers. The third and fourth subsets were used for RT-qPCR and HPLC studies, respectively.

### Behavioral Testing

At the beginning of the 6th week, animals in each group were randomly divided into two subgroups (*n* = 8/subgroup) assigned to different behavioral testing so that each mouse is only tested once per day. Behavioral tests for each subgroup were spaced 1 day apart to avoid any exhaustion or carry-over effects from prior tests.

Animals of the first subgroup were examined for locomotor activity and anxiety-related behavior using the elevated plus maze test, while depressive-like behavior was assessed by the forced swim test. For the second subgroup, the open field test was conducted to assess locomotion and anxiety, whereas depressive-like behavior was evaluated by the sucrose preference and the tail suspension tests. The scheduled test types are represented on the timeline in Fig. 1.

Behavioral tests were performed during the light phase between 07:00 am and 3:00 pm and were videotaped and then analyzed by an observer blinded to the experimental groups. Free access to food and water was allowed all the time except during actual animal testing. No food or water deprivation was applied during the sucrose preference test.

#### Open Field Test

The open field test was performed as previously described [30]. Briefly, a box (60 × 60 × 40 cm) divided into nine equal squares was used. The center square (the central zone) was the starting point for each individual mouse. Animal behavior was observed for 6 min and scored during the last 5 min. The observed parameters were the number of squares crossed (as a measure of locomotion), the number of rearings (as a measure of exploratory behavior), and the time spent in the central zone (as a measure of anxiety). The test apparatus was thoroughly cleaned with 70% ethanol to remove any olfactory cues between animals.

#### Elevated Plus Maze Test

The test apparatus is located 50 cm above the floor and consists of two opposite open arms (5 cm × 25 cm) and two opposite closed arms (5 cm × 25 cm × 25 cm) connected by a central square (5 cm × 5 cm). The central zone facing one of the open arms was the starting point for each individual mouse. The percentage of open-arm entries (open/total entries × 100) and the proportion of time spent in the open arms (open/total time spent in arms × 100) were calculated for each animal during the last 5-min of a 6-min experimental period [31]. The decreased time spent in the open arm or the number of entries reflects more anxiety. After each mouse, the maze was thoroughly cleaned with 70% ethanol to prevent olfactory cues.

#### Sucrose Preference Test

The sucrose consumption test was used to assess the hedonic state, denoting non-depressive behavior [32]. Mice were kept individually in separate cages and were allowed to adapt to two bottles filled with 1.0% sucrose solution for 24 h. For the next 24 h, one bottle of sucrose solution was replaced with water. Then, mice were exposed to two pre-weighed bottles of sucrose solution and water for another 24 h. The position of the bottles was randomly switched to prevent side preference. After the test, the weight of the sucrose solution and water consumed was recorded. Sucrose preference was calculated as a percentage of consumed sucrose solution of the total fluid intake [sucrose preference % = sucrose intake / (sucrose intake + water intake) × 100]. Lower sucrose consumption reflects anhedonia and depressive behavior.

#### Tail Suspension Test

In this test, mice were suspended upside down by their tails 40 cm above the floor by adhesive tape placed 1 cm from the tail tip. During a 6-min test period, the immobility time was recorded in the last 5 min. Prolongation of the immobility time denotes depressive-like behavior [33].

#### Forced Swim Test

Mice were individually placed in a vertical transparent cylinder (30 cm in height and 12 cm in diameter) containing tap water at 25 ± 1 °C and 20 cm in depth for a 15-min training session. The volume of water was enough to prevent the animal from touching the bottom with its tail. After 24 h, a 5-min test session was performed; each mouse was placed in the cylinder, and the duration of immobility (floating with no or minimal movements with head just above the water) was recorded. The greater the time of immobility, the more the mouse is depressed. Water in the cylinder was changed after each mouse [33, 34].

### HO-1 Activity

HO-1 activity was assessed according to the method of Basu et al. [35]. Briefly, a reaction mixture containing 2 mM glucose-6-phosphate (Santa Cruz, USA), 1 U glucose-6-phosphate dehydrogenase, 1 mM NADPH, 25 μM hemin, 2 mg of rat liver cytosolic protein (as biliverdin reductase source), and 100 mM potassium phosphate buffer (pH 7.4) was incubated with 600 μg of brain microsomal protein. The final volume was adjusted to 400 μl with potassium phosphate buffer and incubated for 1 h at 37 °C in the dark. Finally, the reaction was terminated by putting the tubes on ice for 2 min. HO-1 activity was determined via measuring bilirubin concentration as the difference in absorbance between 464 and 530 nm. HO-1 activity was expressed in pmol bilirubin/ mg protein/h.

### Determination of Brain Oxidative and Antioxidant Markers

Hippocampi and cerebral cortices were homogenized in cold PBS (0.1 M, pH 7.4) (1:9, w/v). The homogenate was centrifuged at 12,000 rpm for 10 min at 4 °C. The obtained supernatant was used to estimate MDA and GSH levels and SOD-specific activity, standardized to the brain total protein content estimated by the Lowry method using bovine serum albumin (BSA) (1 mg/ml) [36].

*Malondialdehyde (MDA)* level was estimated using the thiobarbituric acid (TBA) test [37]. One molecule of MDA reacts with two molecules of TBA under acidic conditions with the production of pink color on heating. The absorbance was measured at 532 nm. Brain MDA level was calculated using its extinction coefficient and expressed in μmol/mg protein.

*Reduced glutathione (GSH)* level was measured according to the method of Ellman [38]. GSH reacts with Ellman’s reagent (5,5′-dithio-bis-2-nitrobenzoic acid) producing a yellow-colored 2-nitro-5-thiobenzoic acid product. The absorbance of the developed color was read at 412 nm. The GSH level was expressed as mM/ mg protein.

*Superoxide dismutase (SOD)* activity was assessed via spontaneous autooxidation of pyrogallol at pH 8.2 [39]. SOD enzyme inhibits pyrogallol autooxidation. The change in absorbance was monitored for 2 min spectrophotometrically at 420 nm. A unit of SOD activity is expressed as the amount of enzyme that suppresses 50% of the pyrogallol autooxidation under standard conditions.

### Isolation of RNA and RT-qPCR

Hippocampi and cerebral cortices’ RNA extraction was performed using GENEzol™ Reagent (Geneaid, Taiwan) following the manufacturer’s suggested protocol. RNA yield and integrity were determined by spectroscopy. All the RNA was reverse-transcribed into cDNA using 10 μg total RNA and Viva cDNA synthesis kit (Vivantis, Malaysia). RT-qPCR was performed in triplicate using a reaction mix of 1 μl cDNA template, 10 µl of Taq qPCR Green Master Mix (Vivantis, Malaysia), 2 µl of gene-specific forward and reverse primers (Table 1), and nuclease-free water up to 20 μl. Following denaturation at 95 °C for 2 min, 40 cycles at 95 °C for 15 s, 52/60 °C for 30 s, and 60°for 30 s were performed in 96-well plate format using CFX96™ Real-Time System (BIO-RAD, USA). Fold changes were calculated using the differences in cycle threshold determined by 2^−∆∆Ct^ method. GAPDH was used as an internal reference for the normalization of gene expression.

**Table 1:** The sequence of primers used for real-time PCR analysis and the annealing temperature

Name		Primer sequence	Annealing temp (°C)	Ref
GAPDH	F	AGATCCACAACGGATACATT	52	[40]
	R	TCCCTCAAGATTGTCAGCAA		
IL-1β	F	TGCCACCTTTTGACAGTGAT	52	[41]
	R	TGTGCTGCTGCGAGATTTGA		
TNF-α	F	CCCCAAAGGGATGAGAAGTTC	52	[42]
	R	GGCTTGTCACTCGAATTTTGAGA		
iNOS	F	AAGGACTATCTCCACCAGG	60	[43]
	R	CCTCATGATAACGTTTCTGGC		

### Western Blot Analysis

Briefly, hippocampi and cerebral cortices of sham, OVX, or other treated groups were homogenized in RIPA buffer (50 mM Tris–HCl, pH 8; 150 mM NaCl; 1% Triton X-100; 0.1 SDS; 2 mM EDTA and 1 mM PMSF) supplemented with a protease inhibitor cocktail. A nuclear extraction kit (#ab113474; Abcam) was used to separate nuclear proteins. The homogenates were centrifuged at 14,000 rpm for 20 min at 4 °C. The protein concentration in the total cell and nuclear extract lysates was measured spectrophotometrically using a protein assay kit. Equal amounts of denatured proteins were resolved by 12% SDS-PAGE, transferred to nitrocellulose membranes, and blocked at room temperature for 1 h in a blocking solution containing 5% bovine serum albumin. Membranes containing nuclear proteins were incubated with primary antibodies specific to Nrf-2 (#PA5-27,882, Invitrogen, USA), and Lamin B1 (#13,435 Cell Signaling Technology, USA), while membranes containing total proteins were incubated with primary antibodies for HO-1 (#YPA1919 Biospes, China), BDNF (#YPA1962 Biospes, China), Trx-1 (#2429 Cell Signaling Technology, USA) and β-actin (#4970, Cell Signaling Technology, USA) overnight at 4 °C. The membranes were washed and subsequently incubated for 2 h with goat anti-rabbit alkaline phosphatase-conjugated secondary antibody (#7054, Cell Signaling Technology, USA). The membranes were developed with nitro blue tetrazolium and 5-bromo-4-chloro-3-indolyl-phosphate (NBT/BCIP) solution (Thermo Scientific, USA). Quantification of bands was performed using ImageJ analysis software. β-actin was used as an internal control to normalize the protein expression.

### Determination of Brain Serotonin Levels by HPLC

For HPLC, hippocampal and cortical tissues were homogenized in ice-cold methanol (5 ul/mg tissue weight) and then centrifuged at 14,000 rpm for 20 min. Then, the supernatant was evaporated to dryness by vacuum freezing. The dry residue was further reconstituted with 300 ul deionized water and vortex mixed for 10 s, followed by another 2 min after adding 300 ul of chloroform to isopropanol (100:30, v/v). Following centrifugation at 3000 rpm for 5 min, the upper aqueous layer was separated and injected into the Agilent 1100 chromatography system [42].

Chromatographic separation on a Zorbax SB C18 chromatography column (4.6 × 250 mm, 5 um) was performed by a mobile phase of acetate buffer (12 mM acetic acid, 0.26 mM Na_2_EDTA; pH 3.5):methanol (86:14, v/v). The fluorescence was detected at excitation and emission wavelengths of 279 nm and 320 nm, respectively. Peaks were identified by comparing their retention time in the tissue sample extract solution with that of the standard solution [44].

### Histopathology and Morphometry

Brain tissues fixed in 10% buffered formol saline were processed for routine paraffin block preparation. Coronal brain sections of 5 μm were cut by rotatory microtome, stained with H&E stain, and examined under the light microscope for changes in the hippocampus and prefrontal cortex, the brain regions most affected by anxiety and depression. The thickness of the pyramidal cell layer (PCL) in the hippocampus proper (HP) and the granule cell layer (GCL) in the dentate gyrus (DG) was measured using image analysis software LEICA and the mean value was calculated for each group in high power photos (× 400).

### Statistical Analysis

Data were presented as means ± standard deviation (S. D.) The normality of data distribution was assessed using Shapiro–Wilk’s test. Significant differences between values were analyzed by one-way ANOVA followed by post hoc Tukey’s test. The statistical significance was set at *p* < 0.05. The Statistical Package for Social Sciences 20.0 for Windows (SPSS, Chicago, IL) was used for calculation.

## Results

### Carnosic Acid Ameliorated Ovariectomy-Induced Behavioral Deficits

#### Open Field Test

**Fig. 2:**
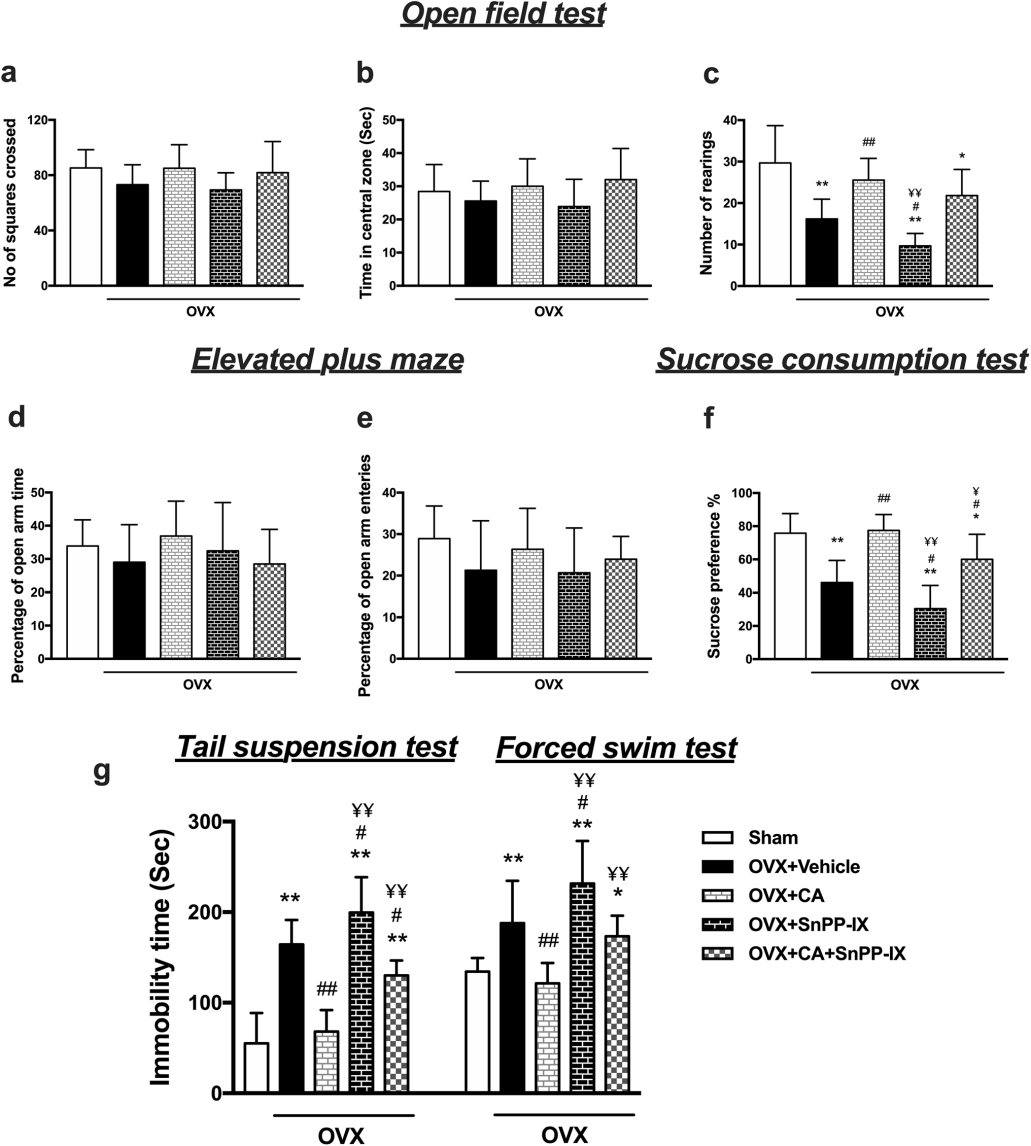
Carnosic acid alleviates depressive-like behavior in ovariectomized mice. Mice were tested for anxiety and exploratory behavior by open field test (**a–c**) and elevated plus maze test (**d**, **e**), and for depressive-like behavior by the sucrose preference test (**f**), tail suspension test, and forced swim test (**g**). Drugs and vehicles were administered starting from the 4th week onward, after OVX, and behavioral tests were performed on the 6th week. The data are presented as means ± S. D., and analyzed by one-way ANOVA followed, when significant, by Tukey’s multiple comparisons test. *p < 0.05 and **p ≤ 0.001 compared with control; ^#^*p* < 0. 05 and ^##^*p* ≤ 0.001 compared with OVX group; ^¥^*p* < 0. 05 and ^¥¥^*p* ≤ 0.001 compared with OVX + CA group. OVX, ovariectomized; CA, carnosic acid; SnPP-IX, tin protoporphyrin IX

On the 6th week after OVX, no significant difference was elucidated among the experimental groups in the number of squares crossed or the time spent in the central zone, implying that ovariectomy did not affect the locomotor activity or induced anxiety-like behavior, respectively. However, OVX mice showed a significant decrease in the number of rearings versus the sham-OVX mice, denoting decreased exploratory behavior which was ameliorated by CA monotherapy as compared to vehicle-treated OVX mice. Combined CA and SnPP-IX treatment was also associated with more rearings, although this increase was not statistically significant compared to OVX mice. Of note, the administration of SnPP-IX alone further decreased the number of rearings compared to the OVX group (Fig. 2a–c).

#### Elevated Plus Maze Test

No significant difference was observed between the experimental groups regarding the percentage of entries or the time spent in the open arms of the elevated plus maze, confirming normal locomotor activity with the absence of anxiety-like behavior among OVX mice (Fig. 2d, e).

#### Sucrose Preference Test

As shown in Fig. 2f, the control mice highly preferred sweetened fluid over tap water, whereas the OVX mice lost interest in sucrose, evidenced by decreased sucrose preference %. This state of anhedonia was ameliorated by CA treatment, where mice showed a high tendency for sucrose consumption similar to control animals. When CA was combined with SnPP-IX, sucrose solution consumption was decreased as compared to the control and CA-treated mice. However, this combined therapy still had some ameliorative effect on anhedonia as compared to the OVX group. On the contrary, SnPP-IX alone decreased sucrose preference % in the OVX mice.

#### Forced Swim Test and Tail Suspension Test

On the sixth-week post-surgery, OVX mice exhibited depressive-like behavior in the forced swim and tail suspension tests reflected as prolonged immobility time compared to the control group. This was normalized by the administration of CA alone with a significant decline in immobility time in both tests relative to OVX mice values. Coadministration of SnPP-IX with CA partially attenuated the potential benefit of CA in alleviating depressive-like behavior as verified by longer durations of immobility. The SnPP-IX-treated mice showed a longer immobility time than the OVX mice (Fig. 2g).

### Carnosic Acid Reversed Ovariectomy-Induced Suppression in Hippocampal and Cortical Expression of Nrf2, HO-1, Trx-1, and BDNF

**Fig. 3:**
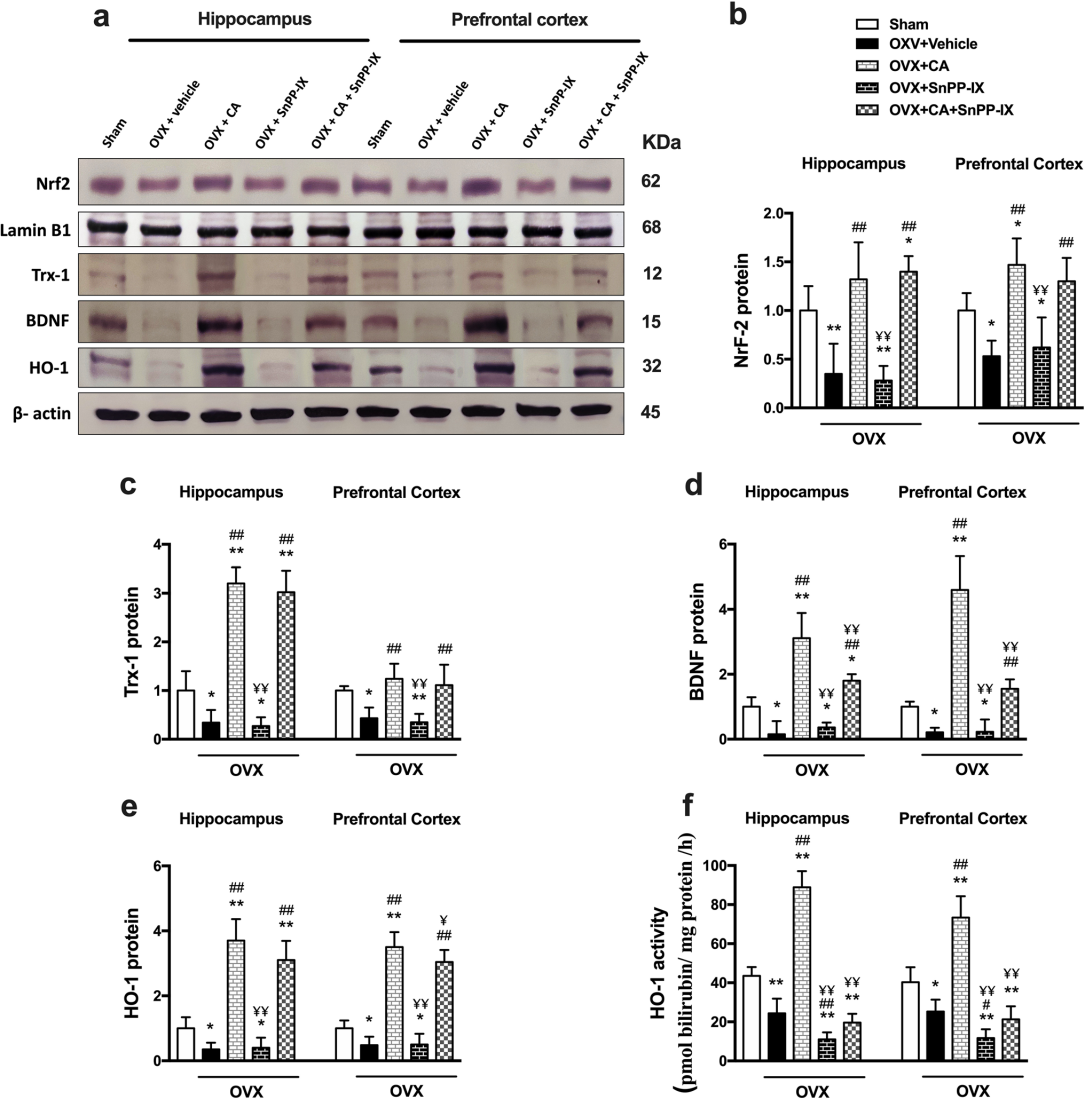
Carnosic acid boosts Nrf2, Trx-1, and BDNF expressions as well as HO-1 expression and activity in the hippocampi and prefrontal cortices of ovariectomized mice. Representative immunoblots for Nrf2, lamin B1, Trx-1, BDNF, HO-1, and β-actin proteins are shown in **a**. The level of nuclear protein expression of Nrf2 was normalized to that of lamin B1 (**b**), while the levels of protein expression of Trx-1 (**c**), BDNF (**d**), and HO-1 (**e**) were normalized to that of β-actin and their relative expression was calculated relative to the control. HO-1 activity (**f**) was determined by measuring bilirubin concentration and expressed as pmol bilirubin/ mg protein /h. The data are presented as means ± S. D., and analyzed by one-way ANOVA followed, when significant, by Tukey’s multiple comparisons test. *p < 0.05 and **p ≤ 0.001 compared with control; ^#^*p* < 0. 05 and ^##^*p* ≤ 0.001 compared with OVX group; ^¥^*p* < 0. 05 and ^¥¥^*p* ≤ 0.001 compared with OVX + CA group. Nrf2, nuclear factor erythroid 2–related factor; Trx-1, thioredoxin-1; BDNF, brain-derived neurotrophic factor; HO-1, hemoxygenase 1; OVX, ovariectomized; CA, carnosic acid; SnPP-IX, tin protoporphyrin IX

As shown in Fig. 3, Nrf2, Trx-1, and BDNF expression levels were significantly decreased in the studied brain tissues of OVX mice when compared with sham-operated mice. The expression of these proteins was upregulated by CA supplementation either solely or when combined with SnPP-IX. Unlike BDNF expression, which was lower in the combined-treated group than in the CA-treated group, Nrf2 and Trx-1 expressions were comparable in these two groups. However, the mice treated by SnPP-IX alone did not show any significant impact on hippocampal or cortical Nrf2, Trx-1, or BDNF protein expression versus the OVX mice (Fig. 3a–d). Regarding HO-1, OVX mice showed depressed expression and activity, while both were stimulated by CA treatment. Notably, administration of SnPP-IX with CA significantly inhibited HO-1 activity in OVX mice compared to mice that received CA alone (Fig. 3a, e, f).

### Carnosic Acid Mitigated Oxidative Stress and Inflammation in OVX Mice

**Fig. 4:**
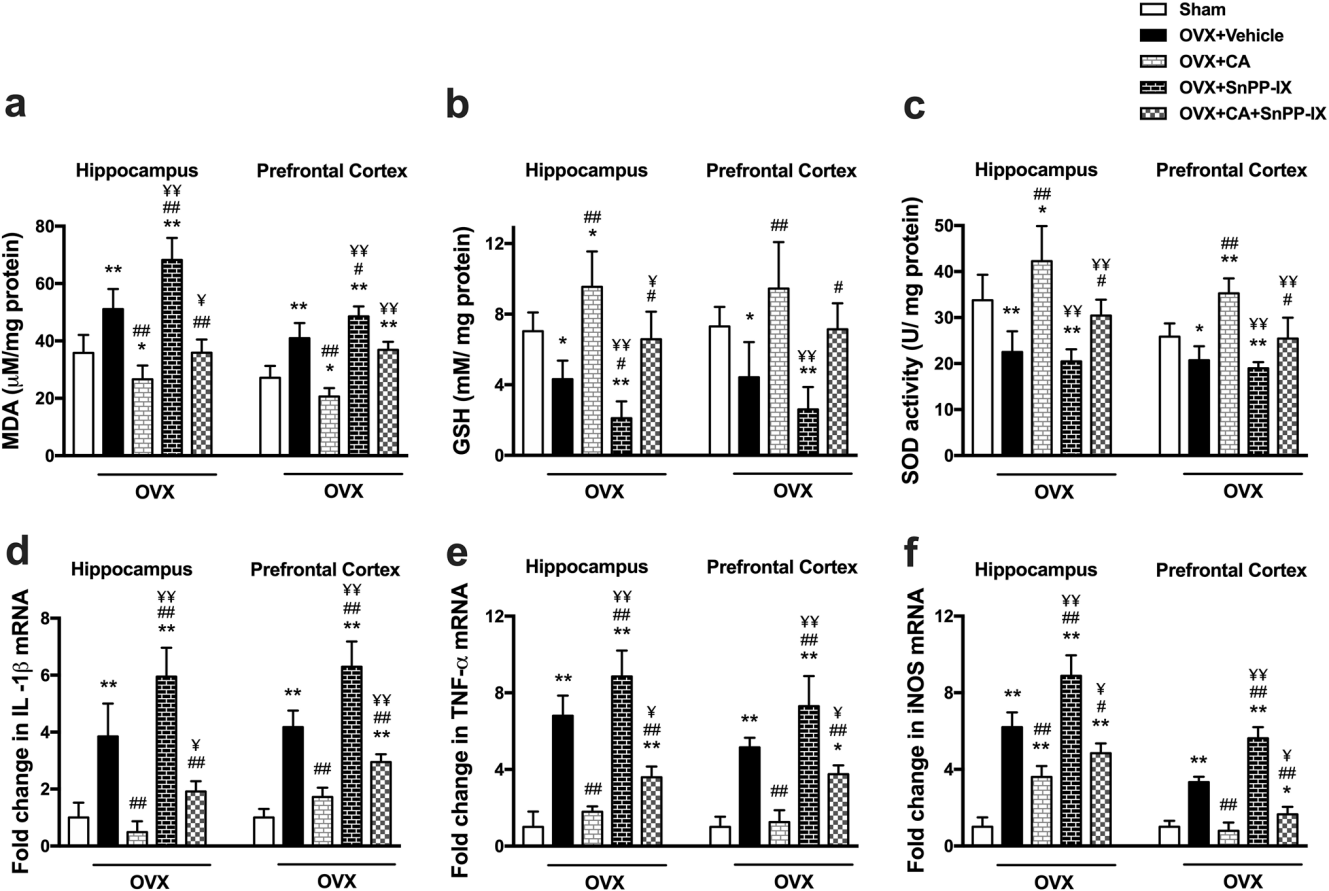
Carnosic acid mitigates oxidative stress and inflammation in the hippocampi and prefrontal cortices of ovariectomized mice. Cellular redox biomarkers including malondialdehyde (MDA), reduced glutathione (GSH), and superoxide dismutase (SOD) activity (**a–c**) were measured by colorimetric method, while the expressions of IL-1β (**d**), TNF-α (**e**), and iNOS (**f**) mRNA were detected by quantitative real-time RT-PCR. The mRNA expression levels were normalized to the housekeeping gene (GAPDH) and expressed as fold changes. The data are presented as means ± S. D., and analyzed by one-way ANOVA followed, when significant, by Tukey’s multiple comparisons test. *p < 0.05 and **p ≤ 0.001 compared with control; ^#^*p* < 0. 05 and ^##^*p* ≤ 0.001 compared with OVX group; ^¥^*p* < 0. 05 and ^¥¥^*p* ≤ 0.001 compared with OVX + CA group. OVX, ovariectomized; CA, carnosic acid; SnPP-IX, tin protoporphyrin IX

Our results revealed that ovariectomy was associated with a significant elevation in MDA and a significant reduction in GSH level and SOD activity in the hippocampus and the prefrontal cortex versus the sham-operated mice. CA monotherapy significantly ameliorated the redox balance where the GSH level and SOD activity increased to values higher than the control, while the MDA decreased to sub-control levels. This powerful antioxidant effect was attenuated when SnPP-IX was combined with CA, although a significant increase in GSH level and SOD activity was observed in the combined-treated group compared to OVX mice. SnPP-IX treatment alone was associated with increased oxidative stress as demonstrated by increased MDA and decreased GSH levels in the cortex and hippocampus of OVX mice (Fig. 4a–c).

Likewise, surgical removal of the ovaries markedly upregulated the expression of proinflammatory genes in cortical and hippocampal tissues as compared to control mice. Such an increase was significantly inhibited in CA and combined-treated groups. However, as shown in Fig. 4d–f, only CA treatment normalized the ovariectomy-induced inflammatory state, except for hippocampal iNOS which was higher than control values. On the other hand, treatment with SnPP-IX was associated with significantly increased proinflammatory mediators versus OVX mice.

### Carnosic Acid Increased Hippocampal and Cortical Serotonin Content in Ovariectomized Mice

**Fig. 5:**
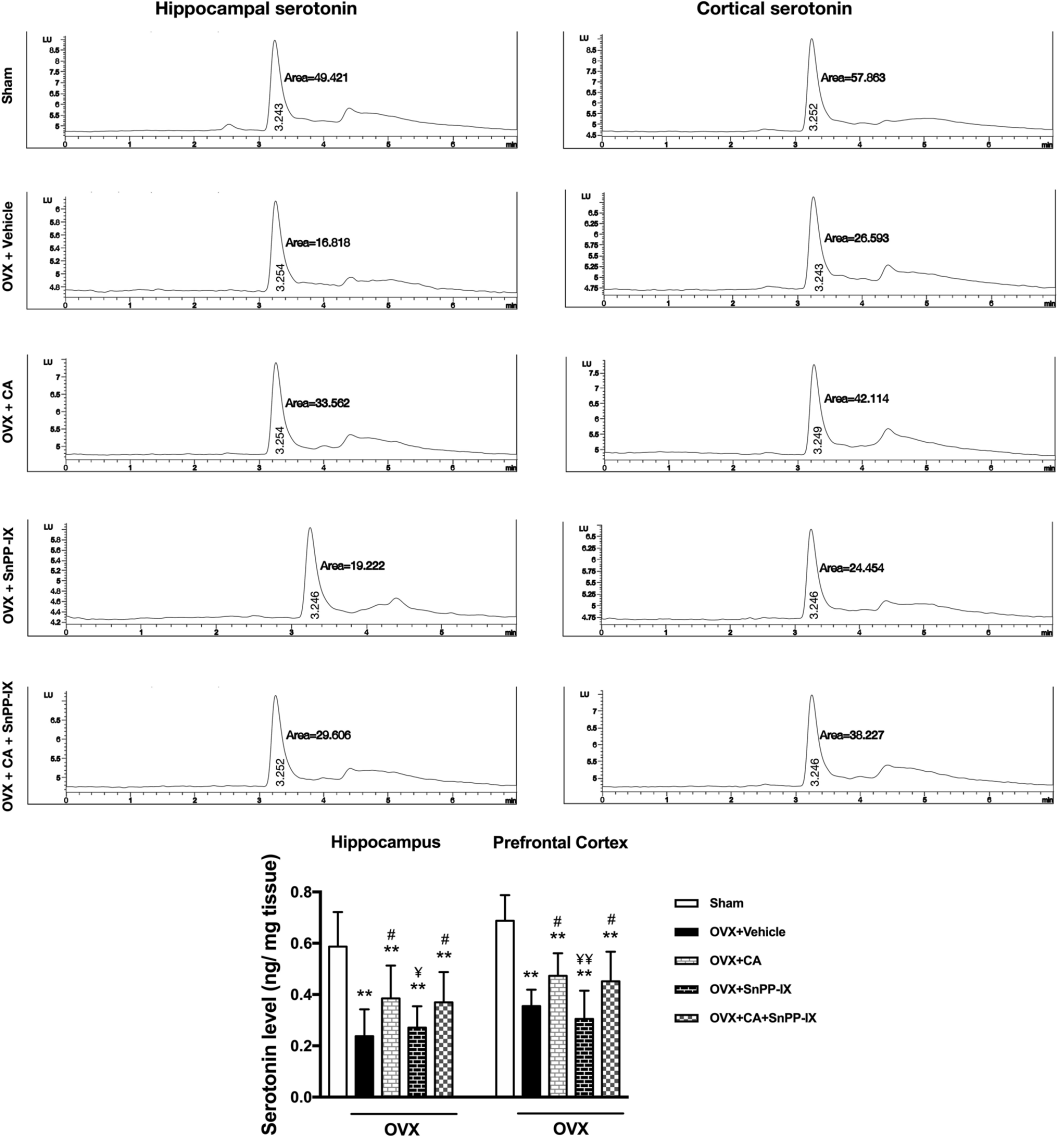
Carnosic acid increases serotonin levels in the hippocampi and prefrontal cortices of ovariectomized mice. Representative graphs for serotonin analysis in hippocampal and cortical tissues by high-performance liquid chromatography (HPLC). The data are presented as means ± S. D., and analyzed by one-way ANOVA followed, when significant, by Tukey’s multiple comparisons test. *p < 0.05 and *p ≤ 0.001 compared with control; ^#^*p* < 0. 05 and ^##^*p* ≤ 0.001 compared with OVX group; ^¥^*p* < 0. 05 and ^¥¥^*p* ≤ 0.001 compared with OVX + CA group. OVX, ovariectomized; CA, carnosic acid; SnPP-IX, tin protoporphyrin IX

Ovariectomy attenuated serotonin levels in hippocampal and cortical tissues as compared to control mice. Administration of CA, alone or combined with SnPP-IX, counteracted such depletion, leading to a significant increment in the serotonin versus the OVX group, though the serotonin level in both groups was significantly lower than that of control mice. Meanwhile, SnPP-IX alone did not alter serotonin levels in OVX mice (Fig. 5).

### Carnosic Acid Alleviated the Histopathological Changes in OVX Mice

**Fig. 6:**
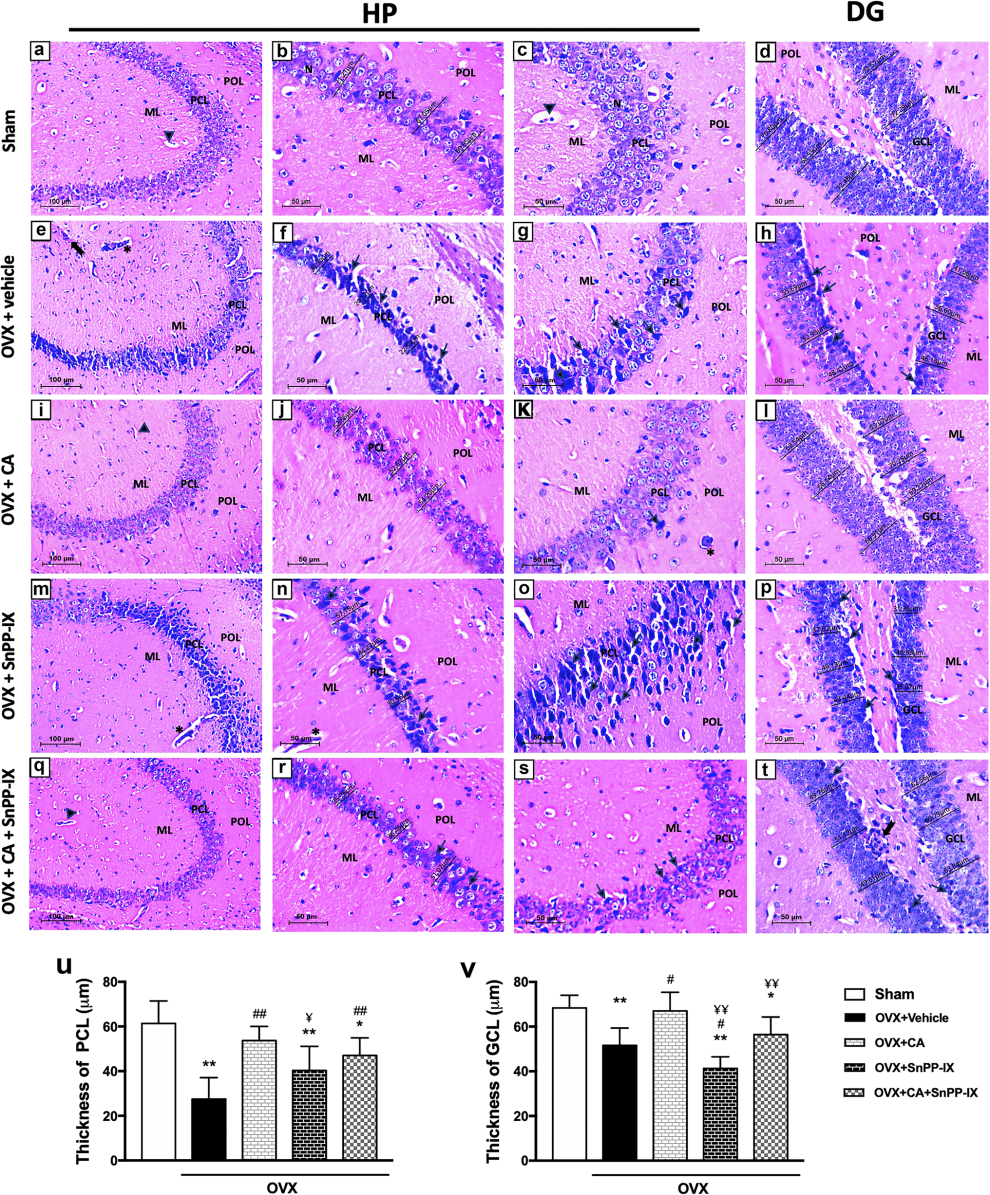
Representative photomicrographs for the effect of carnosic acid and tin protoporphyrin IX given as single or combined treatment on the histopathological alterations detected in H&E-stained sections of hippocampal tissues of ovariectomized mice. The hippocampus of the sham group (**a–d**) shows normal appearance; the hippocampus proper (HP) has three well-defined layers: polymorphic layer (POL), pyramidal cell layer (PCL), and molecular layer (ML). In the PCL, well-organized, closely packed pyramidal neurons (N) are seen with euchromatic nuclei, prominent nucleoli, and scanty cytoplasm. Blood capillaries (arrowhead) are normal. The dentate gyrus (DG), as well, is formed of three layers; ML, granule cell layer (GCL), and POL. In the GCL, aggregation of rounded to oval granule cells is seen. Sections from the OVX mice (**e–h**) reveal thin PCL and GCL in the HP and DG, respectively. Many pyramidal neurons in PCL look shrunken with dark pyknotic nuclei (arrow) and pericellular haloes. Inflammatory cells (bifid arrow) and congested capillaries (*) are seen. Some dark granule cells with pyknotic nuclei were also seen in the DG. The hippocampi of CA-treated group (**i–l**) show almost normal appearance and thickness of the HP and DG, except for congestion of some capillaries (*). Sections from the mice treated with SnPP-IX alone (**m–p**) reveal disorganized and loosely packed PCL. Most pyramidal neurons look shrunken with dark pyknotic nuclei (arrow) and pericellular haloes. Congested capillaries are seen (*). PCL and GCL are thin. Sections of the hippocampus from CA + SnPP-IX group (**q–t**) show moderate thickness of PCL and GCL. Shrunken pyramidal cells and granule cells with dark pyknotic nuclei (arrow) are frequently seen in the HP and DG, respectively. The thickness of the PCL and GCL of the HP and DG, respectively, is measured by using image analysis software LEICA and the mean value is calculated for each group (**u**, **v**). OVX, ovariectomized; CA, carnosic acid; SnPP-IX, tin protoporphyrin IX

Sections from the hippocampi of the sham-operated mice showed the normal histological structure of the two interlocking parts: the hippocampus proper (HP; Cornu Ammonis) and the dentate gyrus (DG) (Fig. 6a–d). Hippocampal sections of OVX mice demonstrated marked neuronal loss in the HP. Many pyramidal neurons were dark and shrunken with pyknotic nuclei and pericellular haloes. Inflammatory cells and congested capillaries were present. The DG was less affected by OVX, and a few dark granular cells were seen (Fig. 6e–h). On the other hand, CA increased cellularity and restored the normal euchromatic appearance of most hippocampal neurons (Fig. 6i–l). Obviously, the histopathological features of the hippocampus deteriorated with SnPP-IX treatment. Most pyramidal cells were irregularly arranged with dark pyknotic nuclei and pericellular haloes. Also, some darkly stained granule cells were found in the DG (Fig. 6m–p). The coadministration of SnPP-IX with CA decreased these neuroprotective responses, where dark shrunken pyramidal cells and granule cells with pyknotic nuclei were frequently seen in the HP and DG, respectively (Fig. 6q–t).

Morphometric analysis revealed a significant thinning of the pyramidal and granule cell layers of the HP and the DG, respectively, relative to normal mice. Supplementation of CA to OVX mice restored their normal thickness, while their thickness was reduced in the group that received SnPP-IX (Fig. 6u, v).

**Fig. 7:**
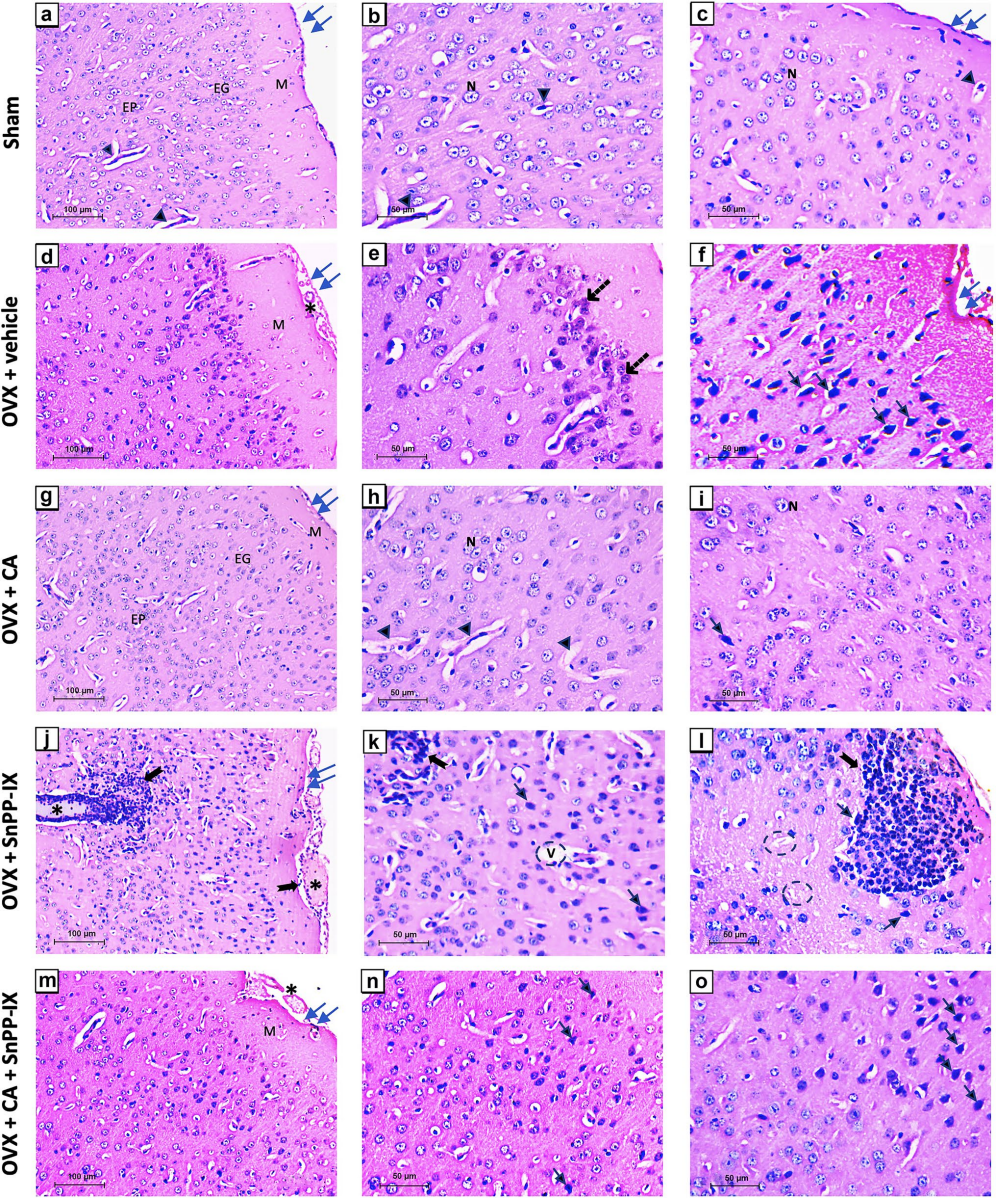
Representative photomicrographs for the effect of carnosic acid and tin protoporphyrin IX given as single or combined treatment on the histopathological alterations detected in H&E-stained sections of the prefrontal cortices of ovariectomized mice. The prefrontal cortex of the sham group (**a–c)** shows normal arrangement of the molecular (M), external granular (EG), and external pyramidal (EP) layers of the cortex. Cell bodies of the neurons exhibit rounded open-face nuclei and prominent nucleoli (N) surrounded with little cytoplasm. Blood capillaries (arrowheads) of different sizes are seen within the eosinophilic neuropil. The cortical surface is regular with well-attached meninges (double arrows). Sections from OVX mice (**d–f**) reveal neuronal cells forming dysregulated cortical laminae with clumped groups of clustered neurons (dashed arrow). The molecular layer (M) is broad. Many neurons are darkly stained and shrunken with pyknotic nuclei and pericellular haloes (arrows). The cortical surface is irregular (double arrows) and shows congested capillaries (*). Sections from CA-treated mice (**g–i)** show neatly arranged cortical laminae with increased number of neuronal cells and increased vascularity (arrowheads). Dark neurons with pyknotic nuclei are rarely seen (arrow). The cortical surface is regular with well-attached meninges (double arrows). Cortical tissues of SnPP-IX-treated animals (**j–l**) show marked cellular infiltration with large patches of inflammatory cells (bifid arrow) and congestion (*). The neuropil appears moth-eaten (circle) or with large vacuoles (v). Cortical laminae are dysregulated and some darkly stained neurons with pyknotic nuclei (arrow) are seen. The surface of the cortex is very irregular (double arrows) and shows congested dilated meningeal vessels (*). In the mice that received combined treatment (**m–o)**, there is a fair arrangement of cortical laminae with broad molecular layer (M). Dark shrunken neurons with pyknotic nuclei (arrows) are frequently seen. The cortical surface is irregular (double arrows) and shows congested capillaries (*). OVX, ovariectomized; CA, carnosic acid; SnPP-IX, tin protoporphyrin IX

Sections from the prefrontal cortices of sham-operated mice showed normal histological structure (Fig. 7a–c), while cortical sections of OVX mice revealed a disrupted arrangement of cortical laminae, and a decreased number of cortical cells, with clumping of some neurons. Numerous dark shrunken neurons with deeply stained pyknotic nuclei were seen. The cortical surface appeared irregular with congested meningeal vessels (Fig. 7d–f). Sections from the prefrontal cortices of CA-treated mice exhibited an almost normal histological appearance, where cortical layers regained normal arrangement. Most neuronal cells appeared normal with rounded euchromatic nuclei and prominent nucleoli, and the meninges were well attached to the regular cortical surface. However, the vascularity was relatively increased (Fig. 7g–i). Intriguingly, a marked infiltration of inflammatory cells was observed in cortical sections from SnPP-IX-treated mice. Furthermore, the cortical surface was very irregular with the separation of the overlying meninges. Meningeal and cortical blood capillaries were markedly congested and dilated. The laminar pattern of cortical neurons was disturbed, and the neuropil appeared vacuolated or moth-eaten. Frequent shrunken cells with pyknotic nuclei were also seen (Fig. 7j–l). Coadministration of SnPP-IX with CA had a negative impact on the prefrontal cortex, where the arrangement of the cortical laminae was distorted, and the cortical neurons were fewer than in CA-treated brains. In addition, meningeal capillaries were congested and dark neurons with pyknotic nuclei were frequently seen (Fig. 7m–o).

**Fig. 8:**
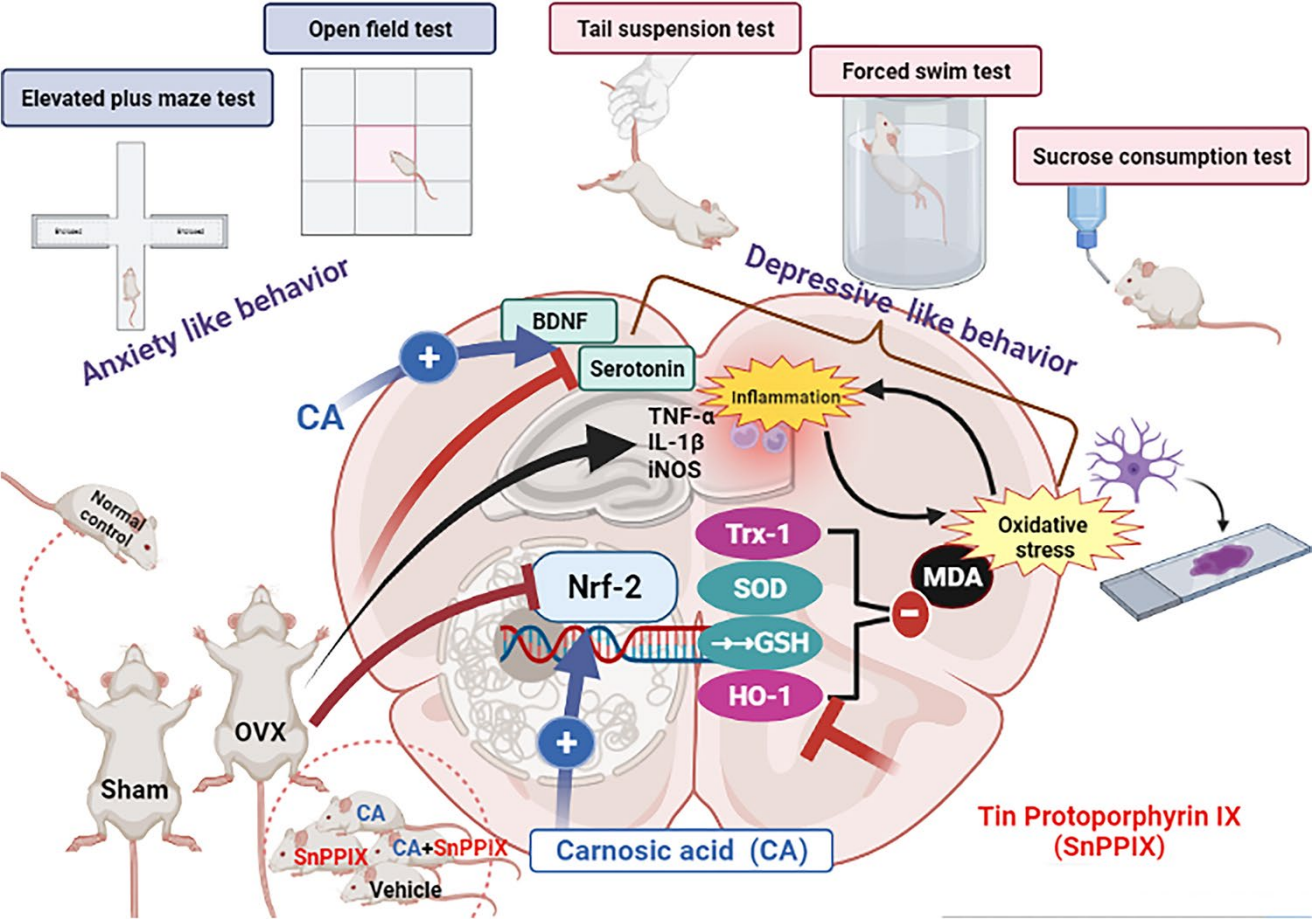
Graphical demonstration of the effect of carnosic acid on the neurobehavior and molecular changes in the hippocampi and prefrontal cortices of ovariectomized mice. Carnosic acid (CA) treatment successfully reverts these changes, while inhibition of HO-1 activity by tin protoporphyrin IX (SnPP-IX) reduces CA-protective effects. OVX, ovariectomized; Nrf2, nuclear factor erythroid 2–related factor; Trx-1, thioredoxin-1; BDNF, brain-derived neurotrophic factor; HO-1, hemoxygenase 1; MDA, malondialdehyde; GSH, reduced glutathione; SOD, superoxide dismutase

A diagrammatic summary of the overall results of the study is presented in Fig. 8.

## Discussion

Depression among peri- and post-menopausal women is an important health problem whose underpinning mechanisms have not yet been elucidated [45]. The fluctuating and declining estrogen levels have been claimed, in part, to affect neurotransmitter synthesis and receptor expression [5]. However, the perturbation of the central redox status secondary to estrogen deficiency is emerging as a centerpiece connecting multiple pathogenic pathways in women’s brains around and after menopause [46]. Nevertheless, the molecular links remained obscure.

The current study provides insights into the potential neuroprotective, antioxidant, and anti-inflammatory effects of CA and its ability to attenuate depressive-like behavior in OVX mice. These beneficial effects were mediated mainly through Nrf2/HO-1 activation as supported by SnPP-IX coadministration.

In this study, OVX mice exhibited a predominant depressive-like behavior as shown by the decreased sucrose preference and the prolonged immobility time in both the forced swim and the tail suspension tests. In contrast, no significant difference in mice performance in either the open field or the elevated plus maze was observed during the sixth week following ovariectomy versus the sham-operated mice, denoting preserved locomotor activity and ruling out any associated anxiety. These results are in line with the studies of Khayum et al. [47] and de Chaves et al. [48], though contradictory to other studies [49, 50]. The differences in the species, age, time from ovariectomy to behavioral assessment, and the conducted tests may explain the discrepancies between the results. On the other hand, the OVX mice reared fewer than the sham-operated mice, reflecting suppressed exploratory behavior that may accompany depression.

Consistent with the disturbed neurotransmitter hypothesis [5], decreased cortical and hippocampal serotonin levels were observed in the OVX mice versus the control mice. In fact, a complex link between estrogen and serotonin has been critically addressed in the literature [51]. Depletion of estrogen may decrease serotonin concentration by losing the inducing effect of estrogen on tryptophan hydroxylase [52], the rate-limiting step in serotonin synthesis, or by losing the inhibitory effect of estrogen on the serotonin reuptake transporter gene expression, thus decreasing serotonin availability in the synapses [53]. Furthermore, with reduced estrogen, 5HT_1A_ receptors are disinhibited, augmenting the negative feedback inhibition of serotonin production [54]. In this study, CA treatment partially regained the OVX-induced serotonin suppression, which may, at least in part, play a role in the encountered antidepressant effect of CA. In concordance, Kumar et al. [55] demonstrated that CA extracted from *Rosmarinus Officinalis L.* increased serotonin levels in rodents’ brains. However, the impact of ovariectomy on serotonin level can only be considered as one contributing mechanism to the encountered depressive behavior. The detected neuronal oxidative, nitrosative, inflammatory, and trophic alterations may also provide tentative alternative explanations for the OVX-induced depressive behavior.

The reactive oxygen species (ROS)–induced oxidative damage was evidently clear by the significantly high level of the oxidative end product, MDA, in the hippocampal and cortical tissues in our study, as well as the high serum MDA level in previous studies [56, 57]. Whereas the observed reduction of GSH can be explained by its rapid consumption in scavenging the excess ROS, the detected decrease in the SOD activity denotes an actual reduction in both enzymatic and non-enzymatic antioxidant defense mechanisms rather than a mere increase in ROS production [58]. Our results clearly indicate that this imbalanced redox state stems from the failure of induction of Nrf2 expression which is the master regulator of the transcription of cellular antioxidant defenses secondary to OVX. Estrogen was recently reported to stimulate Nrf2 activity by a rapid non-genomic activation of the membrane-associated estrogen receptors [59]. To the best of our knowledge, the impact of surgical menopause on hippocampal and cortical Nrf2 expression in relevance to depressive-like behavior was not addressed before. However, a few recent studies have highlighted the defective Nrf-2 expression in the setting of cerebral ischemic injury in OVX rodents [60, 61], which goes in line with our data. The suppressed expression of Nrf2 in OVX mice reduced the induction of many of the downstream antioxidant defensive pathways. Herein, we detected a decreased expression of both hippocampal and frontal cortex HO-1 and Trx-1, contributing further to the redox imbalance.

Since oxidative stress and inflammation are closely related pathophysiological processes, any of them can be easily triggered by the other, perpetuating a vicious circle. We observed an increase in the proinflammatory TNF-α, IL-1β, and iNOS gene expression in both the hippocampus and the frontal cortex, which may explain the inflammation and neuronal loss in the studied brain tissues in our study and in previous studies [62]. In fact, high levels of hippocampal and striatal TNF-α have been associated with anxious and depressed behavior and even preceded the onset of clinical symptoms [63, 64]. Several meta-analyses have shown an increase in serum TNF-α and other proinflammatory cytokines in people suffering from depression [65, 66]. Moreover, IL-1β was reported to suppress in vitro neurogenesis of human hippocampal progenitor cells, a common finding in depression [67, 68]. Overall, inflammation disrupts the BBB, suppresses neurogenesis in the hippocampus, and induces glutamate release from microglia, promoting apoptosis [62, 69]. In line, we observed thinning of the pyramidal and granule cell layers of the HP and DG, respectively, in addition to an increased number of apoptotic cells in the brains of OVX mice versus normal mice.

During the last decades, increasing attention has been directed to phytochemicals due to their pleiotropic activities and low adverse effects [60]. With the significant improvement of all consequences of OVX on the neuronal redox and inflammatory status in addition to serotonin increment in the CA-treated group, this phytochemical seems to be a promising multi-biofunctional antidepressant supplement.

Our data showed that CA treatment ameliorated all depressive-like behaviors, where CA-treated mice showed improved exploration in the open field test and exhibited shorter immobility times in both the forced swim and the tail suspension tests with enhanced sucrose preference versus the vehicle-treated OVX mice. This could, probably, be explained by the observed inducing effect of CA on both hippocampal and cortical Nrf2 expression. The increased Nrf-2 was associated with the restoration of the redox balance, evidenced by the significant reduction of MDA levels. The increased HO-1 and Trx-1 expression and the enhanced SOD activity as well as the high GSH levels indicate that all downstream Nrf2 target genes could be participating in the detected CA-induced cytoprotection, reflected not only in animal behavior but also in brain histology. Mutually, the expression of proinflammatory genes was almost normalized by CA, mitigating the vicious circle between oxidative stress and inflammation.

Interestingly, both hippocampal and cortical BDNF levels were reduced in OVX mice, and such a reduction was reversed by treatment with CA. The observed high level of BDNF may have contributed to the increased cellularity of the prefrontal cortex and thickness of the HP and DG as well as the reduction of darkly stained shrunken neurons in CA-treated animals. A bidirectional relationship between Nrf2 and BDNF has been recently delineated. While some studies pointed to the inducing effect of BDNF on Nrf2 expression [70], others hypothesized the upregulation of BDNF by Nrf2 as an underlying antidepressant mechanism of Nrf2 in rodents [14].

Remarkably, the BDNF was reported to induce the expression of Trx-1 gene. This redox-regulating protein is known to play a major role in controlling cellular ROS by reducing the disulfides into thiol groups [71]. Herein, we found that CA treatment was associated with an increased expression of Trx-1 versus the vehicle-treated OVX mice. Apart from its antioxidant effect, Trx-1 is required for nerve growth factor–mediated signal transduction and neurite outgrowth, and is involved in BDNF-induced synaptic protein expression and in inhibiting neuronal apoptosis [72].

Intriguingly, in this study, a significant reduction of most of the biochemical and cytoprotective effects of CA was depicted when it was combined with the HO-1 inhibitor SnPP-IX, highlighting a fundamental role of Nrf-2/HO-1 axis in CA-induced improvement. This finding further supports the study of de Oliveira et al. [16], who attributed the anti-inflammatory effect of CA to the downstream activation of the Nrf-2/HO-1 axis.

However, the failure of SnPP-IX to completely mitigate the overall cytoprotective effects of CA highlights the fact that other Nrf-2 downstream signaling pathways (such as Trx-1) are also contributing. Furthermore, the proven ability of CA to enhance serotonin levels, apart from HO-1 boosting, in the detected protection cannot be ignored.

Furthermore, the use of SnPP-IX alone was associated with a significant worsening of most of the measured oxidant and inflammatory estimates compared with the OVX mice. This, in turn, may highlight the importance of the residual HO-1 activity post-ovariectomy despite its suppressed expression. Indeed, the function of HO-1 is to catalyze the oxidative degradation of heme to biliverdin, which is transformed into bilirubin under the catalytic effect of biliverdin reductase in the brain and other tissues. Both biliverdin and bilirubin are efficient antioxidants with a high capacity to mitigate oxidative stress and counteract its adverse effects [73].

Accordingly, in our study, the inhibition of HO-1 activity by SnPP-IX, in the absence of the stimulatory effect of CA, resulted in the loss of the antioxidants and survival mechanisms, with subsequent stimulation of proinflammatory gene expression. This was further confirmed by the severe inflammation and neuronal loss detected in the hippocampi and prefrontal cortices of SNPP-IX-treated mice.

## Conclusion

The OVX-induced depressive-like behavior involves major perturbations in the neuronal oxidative and inflammatory milieu in which suppression of Nrf-2 expression is a key mechanism. CA treatment exerted an evident antidepressant activity and restored the distorted redox state. Though activation of the Nrf-2/HO-1 axis by CA was essential, it was not the only contributing mechanism. Upregulation of BDNF and Trx-1 and increasing serotonin in the brain tissue are all interplaying. We, therefore, conclude that CA is an attractive potential phytochemical drug candidate, whose use as an alternative or supplement to traditional antidepressant drugs in post-menopausal depression is worth further clinical evaluation.

## Data Availability

The data of this study are available from the corresponding author upon reasonable request.

## Abbreviations

BDNF: Brain-derived neurotrophic factor
BSA: Bovine serum albumin
CA: Carnosic acid
GCL: Granule cell layer
DG: Dentate gyrus
GSH: Reduced glutathione
HO-1: Heme oxygenase-1
HP: Hippocampus proper
IL-1β: Interleukin-1β
iNOS: Inducible nitric oxide synthase
MDA: Malondialdehyde
Nrf2: Nuclear factor erythroid 2–related factor
PCL: Pyramidal cell layer
ROS: Reactive oxygen species
SnPP-IX: Tin protoporphyrin IX
SOD: Superoxide dismutase
TNF-α: Tumor necrosis factor-α
Trx-1: Thioredoxin-1
